# Encephalitis lethargica in Sweden, 1920–1930: national incidence and the 1921 outbreak in northern Sweden

**DOI:** 10.1017/S0950268826102003

**Published:** 2026-09-08

**Authors:** Lena Karlsson, Erling Häggström Gunfridsson

**Affiliations:** Umeå University, Sweden

**Keywords:** Encephalitis lethargica, Infectious diseases, Sweden, Regional variation

## Abstract

Encephalitis lethargica (EL), also known as the ‘European Sleeping Sickness’, emerged in Europe around 1916, with Sweden reporting the highest global incidence rate in 1921. This study investigates regional variations in EL incidence across Sweden between 1920 and 1930, with a particular focus on the major outbreak in 1921. Using data from *Public Health in Sweden*, incidence rates were calculated for all Swedish regions, revealing a disproportionate concentration of cases in central and northern areas, especially in the parish of Vilhelmina in Västerbotten. To contextualize this outbreak, monthly disease data from the *Annual Reports of the Chief Provincial Doctor in Västerbotten* were analysed to compare the local disease panorama. Notably, preceding the first EL cases in Vilhelmina, influenza/H1N1 and mumps were frequently reported. Although the aetiology of EL remains unresolved, the presence of a preceding influenza and mumps epidemic is consistent with the dual-pathogen hypothesis, suggesting that interactions between infectious agents may have contributed to the emergence of EL in local hotspots.

## Introduction

Encephalitis lethargica (EL), also known as the ‘European Sleeping Sickness’, was a neurological disease that spread globally, originating in Europe around 1916–1917 and continuing into the 1930s and 1940s [1–3]. The first clinical descriptions and reports of EL were made by Constantin von Economo in Vienna on April 17, 1917 [4], although earlier cases had been observed by Jean-René Cruchet during the winter of 1915–1916 among French soldiers in Verdun [5].

Encephalitis lethargica gained significant attention during the 1920s and continues to fascinate researchers across various fields. This enduring interest is largely due to the disease’s sudden emergence, rapid spread, and gradual disappearance, all while its aetiology remains unknown. The condition was marked by devastating neurological and psychiatric symptoms, a high mortality rate during the acute phase, and, in many cases, the later development of post-encephalitic Parkinsonism (PEP) [6–8]. In the wake of the COVID-19 pandemic, there has been a renewed international research interest in encephalitis lethargica. This resurgence is partly due to the fact that both EL and Post-COVID emerged in the context of global pandemics – the Great Influenza of 1918–1919 and COVID-19, respectively. The two conditions share notable similarities, particularly in their psychiatric and neurological manifestations, including post-infectious Parkinsonism, and their suspected links to autoimmune mechanisms [9–13].

### Aim and research questions

Despite Sweden having reported the highest incidence of encephalitis lethargica (EL) cases globally – and a wealth of statistical data collected by public health institutions – there remains a notable lack of research focusing on the extraordinary outbreak that especially affected the northern parts. This study aimed to examine regional variations in EL, with particular emphasis on the disease panorama in northern Sweden during the years surrounding the 1921 outbreak.

The research questions were as follows:

- How did encephalitis lethargica (EL) spread across Sweden between 1920 and 1930?
- Which infectious diseases preceded or coincided with the 1921 EL outbreak in Västerbotten, northern Sweden?
- Are the observed temporal and spatial patterns consistent with existing hypotheses regarding the aetiology of EL?

## Background

### Encephalitis lethargica – Symptoms and epidemiology

The array of symptoms associated with encephalitis lethargica is extensive, encompassing both neurological and psychiatric manifestations. This diversity has made it difficult to establish a clear symptomatology. However, in general, the disease tends to present different symptoms depending on whether it is in the acute or chronic phase. The acute phase included symptoms such as fever, lethargy, blurred vision, headache, and sleep disturbances [14]. In contrast, the chronic phase was characterized by post-EL psychiatric symptoms such as personality changes, psychosis, agitation, and mania, which led to a significant proportion of patients being admitted to psychiatric institutions [14, 15] or by the development of Parkinsonism [16]. According to neurologist Lévy, as cited in Stranger (1925), Parkinsonism was the most frequent residual effect of EL. Of the 129 cases studied, 70 developed Parkinsonism – sometimes just days after the acute onset, and other times months or even years later [14].

Mortality among EL cases appeared to differ between the early years of the epidemic (1919–1920) and the later years, with a significantly higher proportion of deaths occurring during the earlier phase [15]. Furthermore, the age distribution appeared to shift over time. In the early onset of the 1920s, the majority of cases were among younger adults, with 50% of those infected being under 30 years old. In contrast, during the later years of the 1920s, infections became more prevalent among children aged 5–20 years [15]. Regarding gender differences in the incidence of EL, previous findings have been ambiguous. However, Lévy reported a higher incidence among women [17], whereas other case studies have revealed a male surplus [18] or no gender difference [19].

Encephalitis lethargica in children received special attention from contemporary physicians, who described how previously well-behaved and socially functional children underwent dramatic behavioural changes. These included a loss of temper control, regression in cognitive and hygienic skills, and a pronounced restlessness that often disrupted sleep during the night [14, 20]. The psychotic disturbances observed in children were often described as being of a ‘nocturnal type’, where the infected child might attempt to run away from home during the night while appearing more or less ‘normal’ during the day [14]. In particular, the severe psychiatric symptoms observed in infected children – such as aggressive behaviour, personality changes, psychosis, impulsivity, bizarre behaviour, tics, and symptoms resembling obsessive-compulsive disorder – raised significant concerns about how to appropriately treat and manage these children [14, 21]. In the United States, England, and Germany, special schools and behavioural programmes were established to support children affected by EL [21]. For post-encephalitic individuals who were institutionalized in psychiatric facilities, numerous attempts were made to find effective treatments. One notable example is the work of psychiatrist Oliver Sacks, who conducted experimental trials using levodopa. While the drug initially produced dramatic improvements, these effects were often short-lived and eventually followed by regression and deterioration of symptoms [22, 23].

Generally, EL cases were reported across Europe, with a higher incidence observed in urban and industrial areas compared to rural regions [1, 19]. This may have been partly due to the greater availability of medical services and more thorough documentation in urban areas compared to the countryside. Previous studies have also revealed that local outbreaks were common. One of the most frequently cited cases in the literature is an outbreak in a girls’ home in Derby, England, in 1919, where 12 out of 22 pupils were infected, and 5 of them died [24].

It is estimated that between 1917 and 1940, approximately one million individuals were infected with encephalitis lethargica. The average mortality rate was reported to be between 10% and 15%, though it reached as high as 70% in certain outbreaks [15, 25]. The majority of encephalitis lethargica cases were reported in Europe, North America, and Russia [3, 15]. Sweden reported the highest incidence of encephalitis lethargica cases globally, with particularly high concentrations observed in the sparsely populated rural areas of northern Sweden [15].

Even after the 1940s, sporadic cases suspected to be encephalitis lethargica continue to emerge among individuals with no prior medical history [7, 26, 27]. However, there is no clear consensus on whether these modern cases truly represent EL or are instead manifestations of other similar neurological conditions.

### Encephalitis lethargica – The aetiology

*The naked eye appearances of the brain show a characteristic change in colour; it may be a faint pink to a deep salmon colour, and it has been called the rose-coloured brain. On sectioning, the grey matter on the cortex, the basal ganglia, and the cord shows the same change in colour* (Collier and Adie cited at page 4 in [14]).

Since the initial outbreak in 1916–1917, several potential causes of encephalitis lethargica have been proposed. Among these, certain microorganisms have received particular attention – most notably the H1N1 influenza A virus, which is associated with the Spanish Flu [28], herpesvirus [29], poliovirus [30], streptococcus [31], and mumps [32]. More recent studies have proposed alternative causes of encephalitis lethargica, including arboviruses [33] and enteroviruses as potential culprits [34].

Regarding the most commonly proposed cause of EL – the H1N1 virus – Ravenholt and Foege [25] compared death records from Western Samoa, which was severely affected by the influenza pandemic, with those from American Samoa – which largely escaped the influenza pandemic due to its rapid quarantine. They found only two deaths attributed to encephalitis lethargica in American Samoa, compared to 78 in Western Samoa. Based on these findings, the authors concluded that EL and post-encephalitic Parkinsonism were consequences of the 1918–1919 influenza pandemic [25]. While in some regions EL appeared to move in parallel with the Spanish Flu, in others it either preceded or followed the pandemic by several years, weakening the argument for a direct causal link. Moreover, whereas the Spanish Flu spread from the United States to Europe, EL appeared to spread in the opposite direction – from Europe to the United States [35]. Laboratory studies attempting to detect influenza RNA in archived brain tissue from EL patients have failed to find evidence that the influenza virus acted as a neurotropic agent in these cases [3, 35]. A range of viral candidates has been investigated in archival brain tissue from encephalitis lethargica cases, including reports of enterovirus-like particles in affected neurons [34]; however, these findings are based on a small number of cases (*n* = 4) and have not been replicated.

Environmental factors – such as exposure to toxins – or the hypothesis of encephalitis lethargica being a vector-borne disease have also been investigated and largely ruled out as plausible causes. This conclusion is supported by the fact that the majority of EL cases occurred during the winter months in the Northern Hemisphere, a period when vector activity is typically minimal [3, 35].

Taken together, the proposed causes and mechanisms of encephalitis lethargica can be broadly categorized into three main hypotheses. The first is the monocausal hypothesis, which suggests that a single virus or bacterium – either known or yet unidentified – acted independently of other pathogens to cause the disease [7, 35]. Among the known pathogens, H1N1 is the most plausible candidate. In this view, even in the absence of detectable influenza RNA in brain tissue, a connection cannot be entirely excluded. One possible explanation is that encephalitis lethargica represents a post-infectious process, with H1N1 as a principal aetiological factor, analogous to the association between COVID-19 and post-COVID syndrome [35, 36]. Other plausible candidates for triggering a post-infectious process consistent with EL are streptococcus [7], mumps [32], and enteroviruses [34]. Regarding epidemics occurring concurrently with encephalitis lethargica (EL), which may have been associated with it, mumps has received some attention. The Norwegian military physician Gundersen noted that encephalitis lethargica appeared to coincide with, or ‘ride on the crest’ of, the mumps epidemic waves in Oslo [32]. Peaks in encephalitis lethargica cases in December 1919 coincided with what he described as ‘the worst epidemic of parotitis in progress’, a pattern that appeared to recur the following winter in 1920 [32]. The close temporal spacing of the mumps epidemics in 1919 and 1920 may indicate increased epidemic activity and possibly greater virulence of the parotitic infection. Although mumps was generally classified as a childhood disease, Gundersen regarded it as polymorphous, with epidemics varying in symptoms and age distribution, sometimes predominantly affecting adult individuals. Gundersen was not alone in suggesting a possible association between encephalitis lethargica and mumps; von Economo reported parotitis as a prodromal feature in some cases of encephalitis lethargica [37].

The dual hypothesis that proposes that encephalitis lethargica was caused by the interaction between two pathogens – either viruses or bacteria – working in tandem. For example, a known virus such as the influenza virus may have weakened the immune system or altered the host environment in a way that allowed a second, unidentified or known pathogen to trigger EL. In this model, a primary infection potentiates the effects of a subsequent infection, reducing host resistance and facilitating EL onset; as in the monocausal hypothesis, this may lead to a post-infectious reaction [20, 23]. Further, viruses can circulate simultaneously in epidemic form, as has been observed both historically– for example, the H1N1 influenza and typhus outbreaks in Europe– and in contemporary settings, such as the co-circulation of COVID-19 and seasonal influenza, potentially influencing the disease response [36].

The autoimmune hypothesis, represented by more recent research, has emphasized the possibility that encephalitis lethargica may have been an autoimmune disorder, triggered by a viral or bacterial infection. In this model, the initial infection does not directly cause the neurological symptoms but instead activates an abnormal immune response. This response may lead the body to mistakenly attack its own neural tissues, resulting in the characteristic symptoms of EL. This hypothesis is supported by parallels with other post-infectious autoimmune conditions and by emerging evidence of immune-mediated mechanisms in some modern encephalitic syndromes, such as Pediatric Acute-Onset Neuropsychiatric Syndrome (PANS) [7, 35]. The autoimmune process may be triggered by various pathogens. Studies have suggested that streptococcus bacteria [7, 35] or influenza viruses (e.g. H1N1) [3] could initiate an abnormal immune response. Furthermore, the autoimmune response may have resulted from the simultaneous presence of two pathogens – either viral or bacterial – that acted synergistically to trigger the immunological reaction leading to encephalitis lethargica [38].

### The research area – encephalitis lethargica in Sweden

*So formed* e.g. *the population of Vilhelmina such a virgin land, where the infection, once penetrated, made itself felt through numerous more severe cases….* [Kling & Liljenquist 1921:923]. Original citation from Kling & Liljenquist (1921:923) at page 8: ‘Så bildade t ex. befolkningen i Vilhelmina en sådan jungfrulig mark, där smittan en gång inträngd gav sig till känna genom talrika svårare, med nervsymtom sig manifesterade fall’.

The first EL cases in Sweden were diagnosed and described by the physician Nils Antoni in Stockholm during the autumn of 1919 [39]. Antoni strongly emphasized that EL was caused by an unknown virus [38]. Compared to all other countries, Sweden had the highest reported incidence rate of EL, with epidemics during the winters of 1920/1921, 1921/1922, and 1922/1923 [15]. The winter of 1920–1921 marked the peak of European EL cases, including in Sweden [15]. In 1921, 1,512 cases were reported nationwide, including 187 in the rural parish of Vilhelmina in northern Sweden. This pattern contrasts with most epidemic infectious diseases during the industrialization era, which tended to affect urban areas to a much greater extent [40]. Furthermore, compared to other countries, Sweden appeared to report a higher number of mild or abortive cases of EL [15].

One of the earliest reports on the EL epidemic in Sweden was a study conducted by pioneering polio researcher Carl Kling and his colleague Folke Liljenquist at the National Swedish Bacteriological Laboratory. Their work focused on the outbreak in Vilhelmina, located in Lapland [41]. The investigators visited four hamlets in Vilhelmina – Lövliden, Nordansjö, Skansholm, and Skog – between February 4 and February 12, 1921 [41]. The results of Kling and Liljenquist’s study revealed a point prevalence of 27% across the four hamlets, with 35 EL cases identified in a population of 129 individuals [38]. The investigators described the symptoms of the EL cases in Vilhelmina as beginning with an initial episode of fever, severe headache, vertigo, insomnia, and body aches. After a few days, these symptoms progressed into severe fatigue, a sensation of heaviness in the head, intense scalp pain, hallucinations, and blurred or double vision [41]. The symptoms observed in the EL cases in Vilhelmina, as described by Kling and Liljenquist in 1921, were largely consistent with those reported in other studies and case reports from the same period. However, the Vilhelmina cases more frequently exhibited mild upper respiratory tract symptoms – such as nasal congestion or a mild cough – during the initial days of illness [15].

Ruling out the possibility of vector-borne transmission (e.g., lice or mosquitoes), as well as transmission via food, water, or other environmental factors, Kling and Liljenquist concluded that encephalitis lethargica was transmitted from person to person and that ‘…one may trace its gradual and unfolding expansion along the human pathways’ (p. 127) [41]. Their conclusion contradicted the prevailing view at the time, as there was no clear evidence supporting person-to-person transmission of EL [3]. Furthermore, Kling and Liljenquist estimated the incubation period to be 3–10 days, suggested that an infected individual remained contagious for 10 days after infection, and proposed that the cause was most likely an unknown pathogen [41]. Their findings were supported by observations that multiple members of the same household were infected, and that several visitors to Vilhelmina became ill upon returning home to Kittelfjäll (150 km away), subsequently infecting members of their own households [41]. They also noted that the lower incidence of EL in towns and larger cities, compared to rural areas, could be explained by higher levels of immunity to the unknown virus in the more densely populated urban populations [41].

The outbreak in Vilhelmina, a province in northern Sweden, during the winter months of 1921 attracted international interest among researchers and physicians – primarily at the time [16] but also in more recent studies [38, 42]. The Vilhelmina case was considered rather unique, not only due to its high point prevalence but also because of the unusually high number of abortive cases (mild febrile episodes) among household members. This pattern suggested that encephalitis lethargica may have been an occasionally severe clinical manifestation of a more widespread, typically mild infection [42].

## Data and method

### Statistical sources and methods

Encephalitis lethargica was classified as a notifiable disease in Sweden from July 1920 to 1953 when the ‘Epidemic act’ was replaced by the ‘New Epidemic act’ [38]. This required physicians to notify health authorities of diagnosed cases, making the available statistics more complete and reliable than for many other infectious diseases during the same period. Data on reported EL cases – categorized by region, month, and urban versus rural areas – were collected from the annual report Public Health in Sweden (1920–1945), published by the Swedish Royal Medical Board (Kungliga Medicinalstyrelsen) [43]. To compare the incidence rate of encephalitis lethargica (EL) across Sweden’s administrative regions, population data for each region were obtained from the annual Statistics Sweden Population in Administrative Districts, 31 December (1910–1961) [44].

To compare the disease panorama in Västerbotten County, the Annual Reports of the Chief Provincial Doctor in Västerbotten (Årsberättelser från förste provinsialläkaren i Västerbotten) have been utilized. These reports include not only population figures for each province but also detailed accounts of the region’s public health status. They provide monthly data on the number of reported cases for approximately 15 infectious diseases, as well as cause-of-death statistics disaggregated by gender and age group. The reports are archived at the Regional Archive of Västerbotten in Sweden. They have been digitized and imported into the statistical software package R for data analysis and map-based visualization. Incidence rates were calculated as the number of EL cases per 100, 000 inhabitants.

### The research area

For spatiotemporal distributions of EL – the total population of Sweden was included in the analyses. Sweden’s population in 1921 was approximately 5.9 million people where about 65% lived on the countryside [45]. In 1921, the population of Västerbotten was approximately 185,000. This population included two urban centres, Skellefteå and Umeå, with 3,000 and 7,500 inhabitants, respectively. The county was administratively divided into 20 rural districts, with population sizes ranging from approximately 4,000 to 28,000 inhabitants. Vilhelmina constituted the sixth largest district, with a population of 9,575 (see Figure 1).

**Figure 1.**
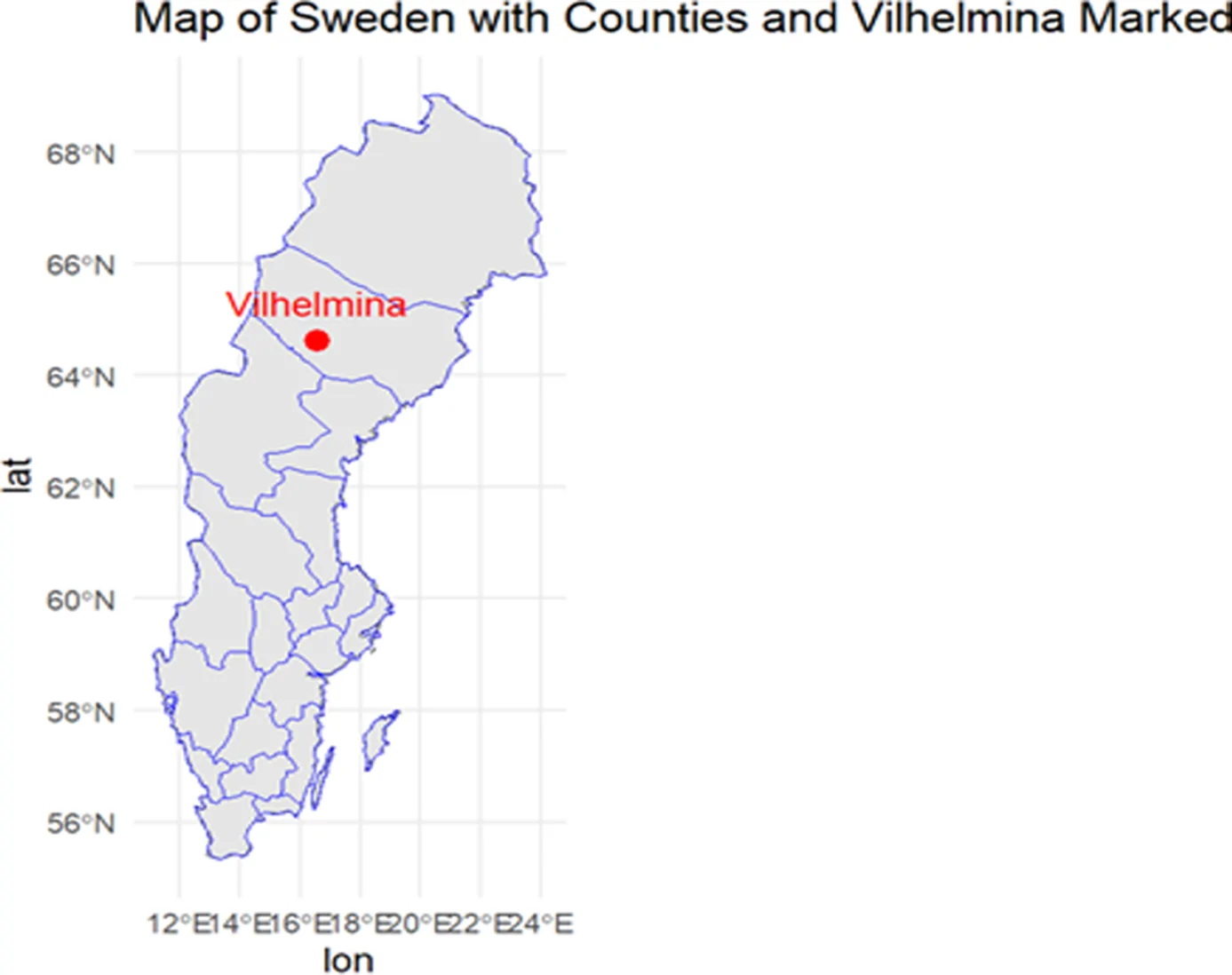
Map of Sweden with Vilhelmina marked.

### Infectious diseases

Building on the main hypotheses proposed in previous research regarding the aetiology of encephalitis lethargica this study compared the incidence of EL with that of other infectious diseases in Västerbotten during the years 1920 and 1921. The following infectious diseases from the Annual Reports of the Chief Provincial Doctor in Västerbotten were included in the comparative analysis: *Scarlet fever* [streptococcus], *Mumps, Influenza/Spanish Flu* [H1N1], *Diphtheria, Measles*, and *Pertussis/Whooping cough.*

Sweden has a long tradition of official statistics, which are generally considered to be of high quality and have been widely used in research on historical epidemics, particularly the Spanish influenza. However, some limitations should be noted. First, the lack of age-specific data for infected individuals restricts detailed comparisons between encephalitis lethargica (EL) and other infections, as age distributions are only available for mortality data for this study period. Second, the set of notifiable diseases varied over time, reflecting shifting national priorities. In addition, reporting practices differed between provincial doctors for non-notifiable diseases. Consequently, conditions such as mumps were not systematically recorded at the national or regional level and may only appear in local sources (e.g. physicians or hospital records).

## Results

In contrast to other heavily affected countries – such as the United States and England, where encephalitis lethargica peaked around 1923 [46] – Sweden experienced its highest number of reported cases in 1921 (see Figure 2). This was followed by a smaller peak in 1923, after which the number of EL cases gradually declined, with only sporadic cases reported by the mid-1940s. Mortality was highest in 1921, with 370 deaths, and between 1920 and 1930, a total of 1,424 deaths due to encephalitis lethargica were reported in Sweden [47].

**Figure 2.**
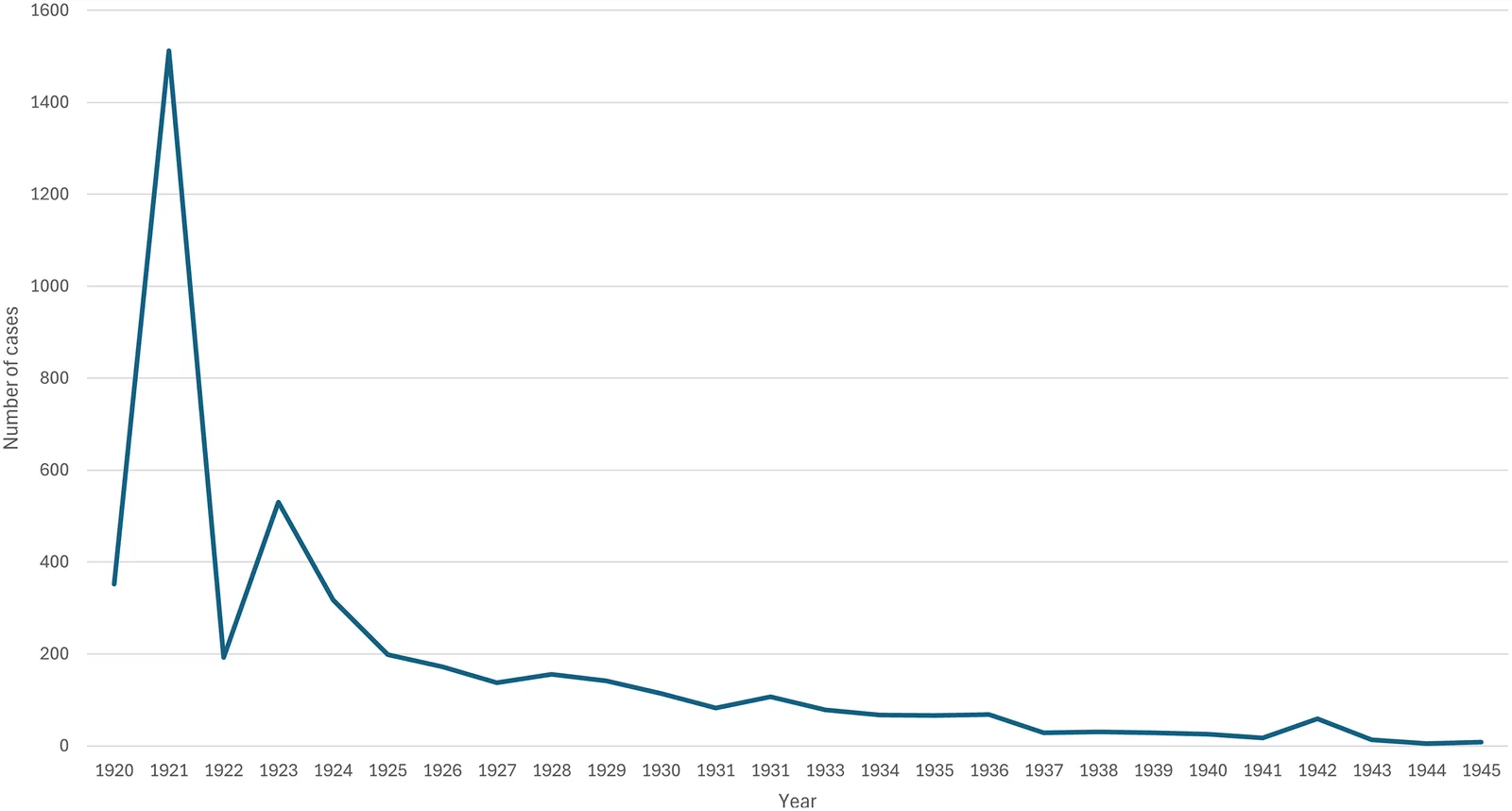
Number of reported encephalitis lethargica cases in Sweden 1920–1945. Sources: Statistics Sweden. Figure 2. long description.

Figure 3 presents the incidence rates (cases per 100,000 inhabitants) of reported encephalitis lethargica cases across Swedish regions between 1919 and 1930. For 1919 and 1920, the rates are limited because EL was only introduced as a notifiable disease on July 1, 1920. The major cities of Sweden – Uppsala, Stockholm, Gothenburg, and Malmö – are marked with red rings on the maps. These visualizations reveal that EL primarily affected populations in medium-sized towns and rural areas rather than in the largest urban centres. In 1920, the highest incidence rates were observed in the East Middle and East Northern regions of Sweden, including the capital Stockholm (30 cases) and the surrounding Stockholm region (31 cases). During the major outbreak in 1921, all regions in Sweden – except the city of Malmö – reported EL cases. However, the cases were heavily concentrated in the central and northern parts of the country. That year, Västerbotten had the highest incidence rate (131 cases per 100,000), followed by Gävleborg (101), Västernorrland (79), and Kopparberg (67). In 1922, there was a sharp decline in EL incidence and a movement from North to South, followed by a modest resurgence in 1923 and 1924, with a concentration in the West Middle and West Northern regions. From 1925 to 1930, no major outbreaks were recorded in any region. However, the Kalmar region in southern Sweden reported 29 cases in 1928 – its highest incidence during the entire 1919–1930 period.

**Figure 3.**
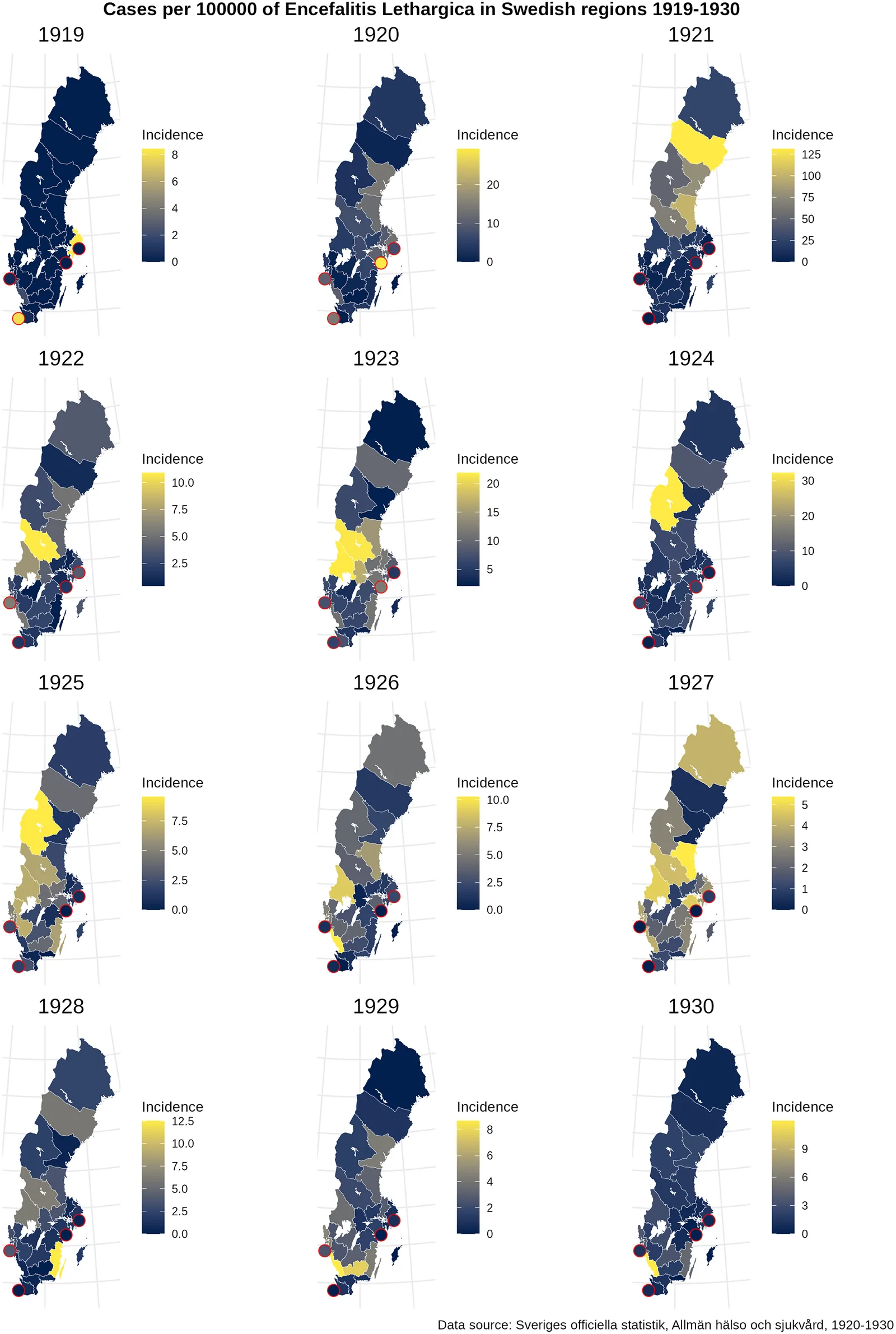
Encephalitis lethargica incidence (per 100,000) in Sweden 1919–1930 by administrative region. Figure 3. long description.

Overall, during the entire period from 1919 to 1930, encephalitis lethargica was primarily concentrated in the central and northern parts of Sweden. Based on the annual reports that distinguish between reported EL cases in urban and rural areas, the impression that EL predominantly affected rural populations is confirmed. Between 1921 and 1945, a total of 3,248 EL cases were reported among individuals living in the countryside, compared to 908 cases in Sweden’s largest cities (data not shown in tables). Unfortunately, the reports do not provide information on the urban–rural distribution of EL cases for the year 1920, when the disease first became notifiable. In 1921, the incidence rate in rural areas was 32 per 100,000, compared with 10 per 100,000 in urban areas. Further, encephalitis lethargica exhibited a clear seasonal pattern, with higher incidence rates during the winter months, particularly from January to March (Figure 4). This seasonal trend corresponds to that of most upper respiratory infections, such as influenza and other viral respiratory illnesses.

**Figure 4.**
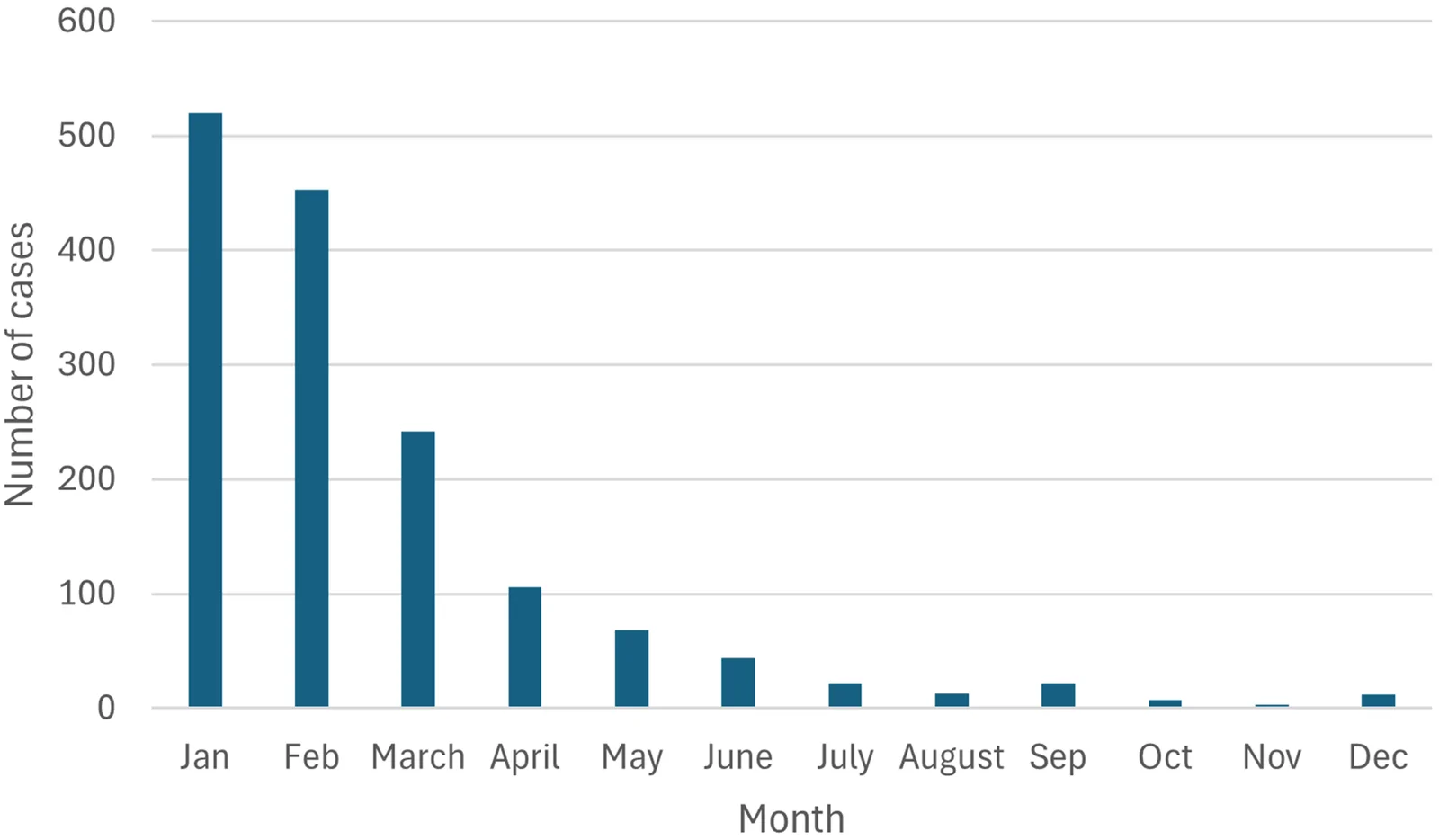
Reported encephalitis lethargica cases in Sweden 1921 by month (per cent). Figure 4. long description.

### Encephalitis lethargica and the disease panorama in Västerbotten 1920–1921

The first reported case of encephalitis lethargica in Västerbotten County occurred in January 1920 in Skellefteå, the county’s second-largest municipality, located along the coast (population approx. 25,000). Eleven months later and when EL has become a notifiable infection, a second case was reported in the small inland municipality of Bjurholm (population approx. 5,800). In January 1921, eight municipalities in Västerbotten reported EL cases, with Bjurholm recording the highest number (5 cases). A significant increase followed in February 1921, when Vilhelmina reported 130 cases, followed by Stensele and Nordmaling, each with 9 cases (results not shown in table). Of the total 243 EL cases reported in Västerbotten in 1921, 77% (187 cases) were concentrated in Vilhelmina.

Based on the disease panorama in Västerbotten from January 1920 to December 1921, Figure 5 illustrates the high number of influenza cases during the winter and spring months, particularly from January to May 1920. Among all reported diseases, influenza – including the Spanish flu – was the most widespread infection during the first half of 1920, followed by diphtheria. From July 1920 onwards, mumps began to spread rapidly, peaking in December and becoming the most prevalent infectious disease in Västerbotten by the end of the year. In early 1921, upper respiratory infections such as influenza and whooping cough increased again. However, in February 1921, encephalitis lethargica accounted for the highest number of reported cases, and it remained the second most common infection in March. After that, only isolated or sporadic EL cases were reported until October.

**Figure 5.**
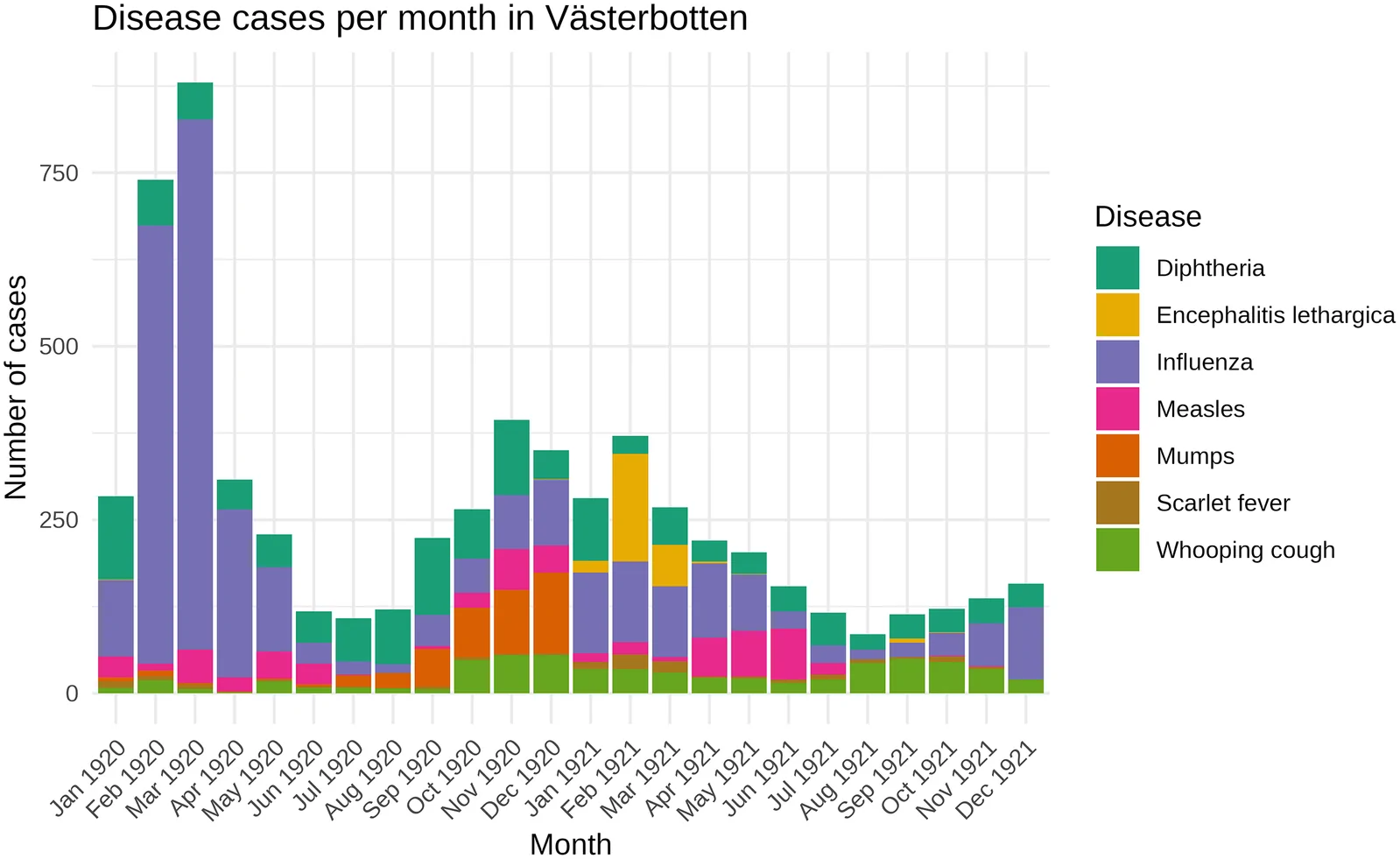
Number of infected individuals by disease and month reported in Västerbotten 1920–1921. Figure 5. long description.

#### Vilhelmina 1920–1921

As in most counties, influenza was the most widespread infection in Vilhelmina during the first 5 months of 1920 (incidence 1527/100000), followed by diphtheria (incidence 237/100000) (Figure 6). From July to December 1920, 361 cases of mumps were reported in Vilhelmina (incidence 3778/100000), compared with a total of 373 mumps cases in the entire county of Västerbotten that year. Controlling for other infections reported between July and December 1920 in Vilhelmina, there were simultaneous reports of ‘Acute inflammation of the trachea’ (*n* = 109) and ‘Pneumonia’ (*n* = 13).

**Figure 6.**
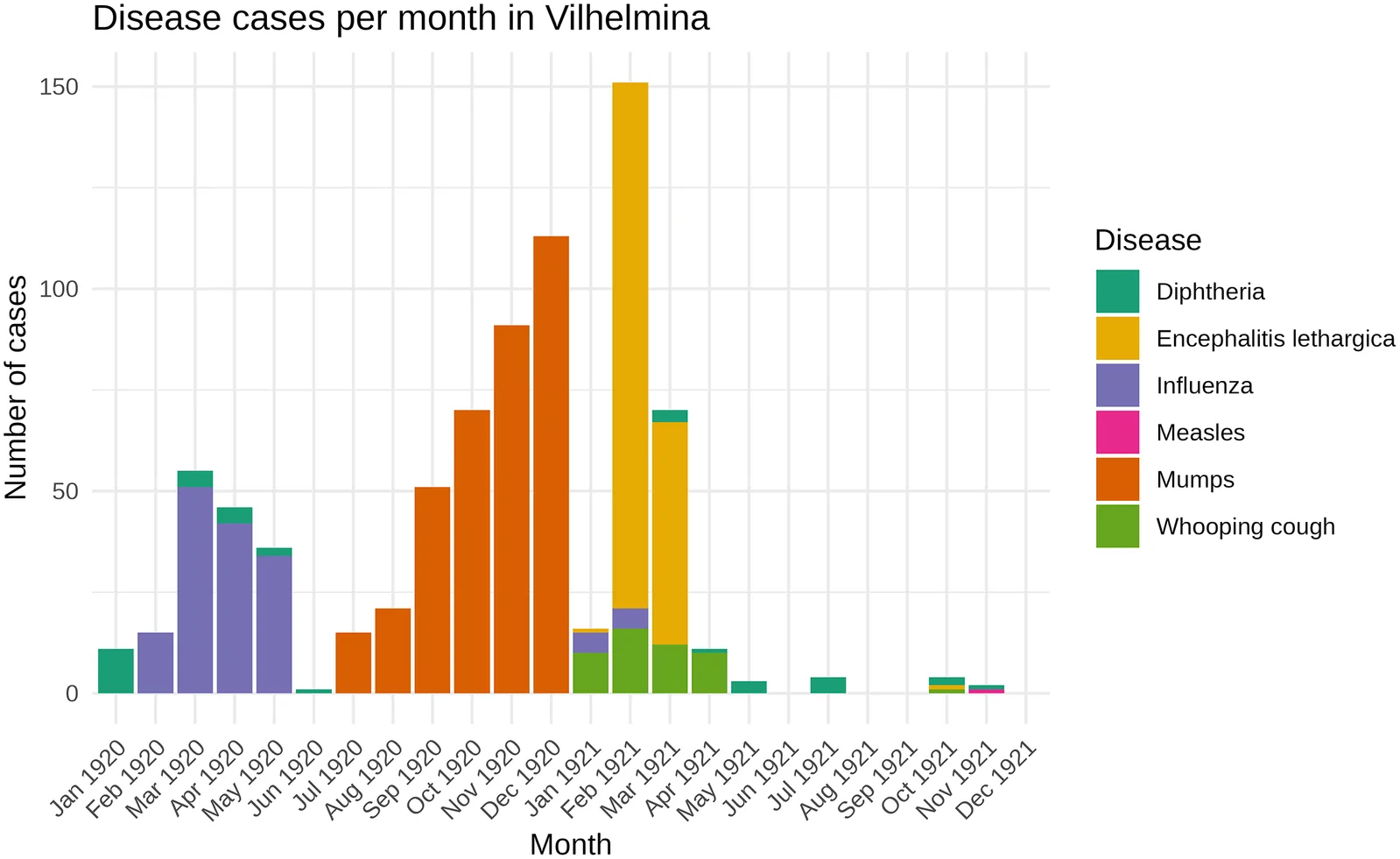
Number of reported infected individuals in Vilhelmina by disease and month, 1920–1921. Figure 6. long description.

Of the 187 EL cases reported in Vilhelmina in 1921 (incidence 1957/100000), 185 cases were reported between February to March along with isolated cases of whooping cough and influenza. Unfortunately, from 1921 onwards, mumps was no longer a notifiable disease, and provincial doctors were therefore not required to report cases. As a result, the temporal relationship between mumps and encephalitis lethargica can only be examined for the period when both infections were subject to mandatory reporting.

### Mumps and EL incidences in Sweden 1920

Incidence comparisons between the two infections are therefore restricted to periods during which both were notifiable, specifically the year 1920. Although, plotting the incidence rates for EL and mumps in Sweden 1920 (Figure 7) shows a weak correlation (Pearsons R2 0.27), interestingly is that the Norrköping region (the second smallest region in Sweden during that time) had the markedly highest incidence of mumps (308/100000) and also reported the noticeable highest number of EL cases that year (incidence rate 29/100000).

**Figure 7.**
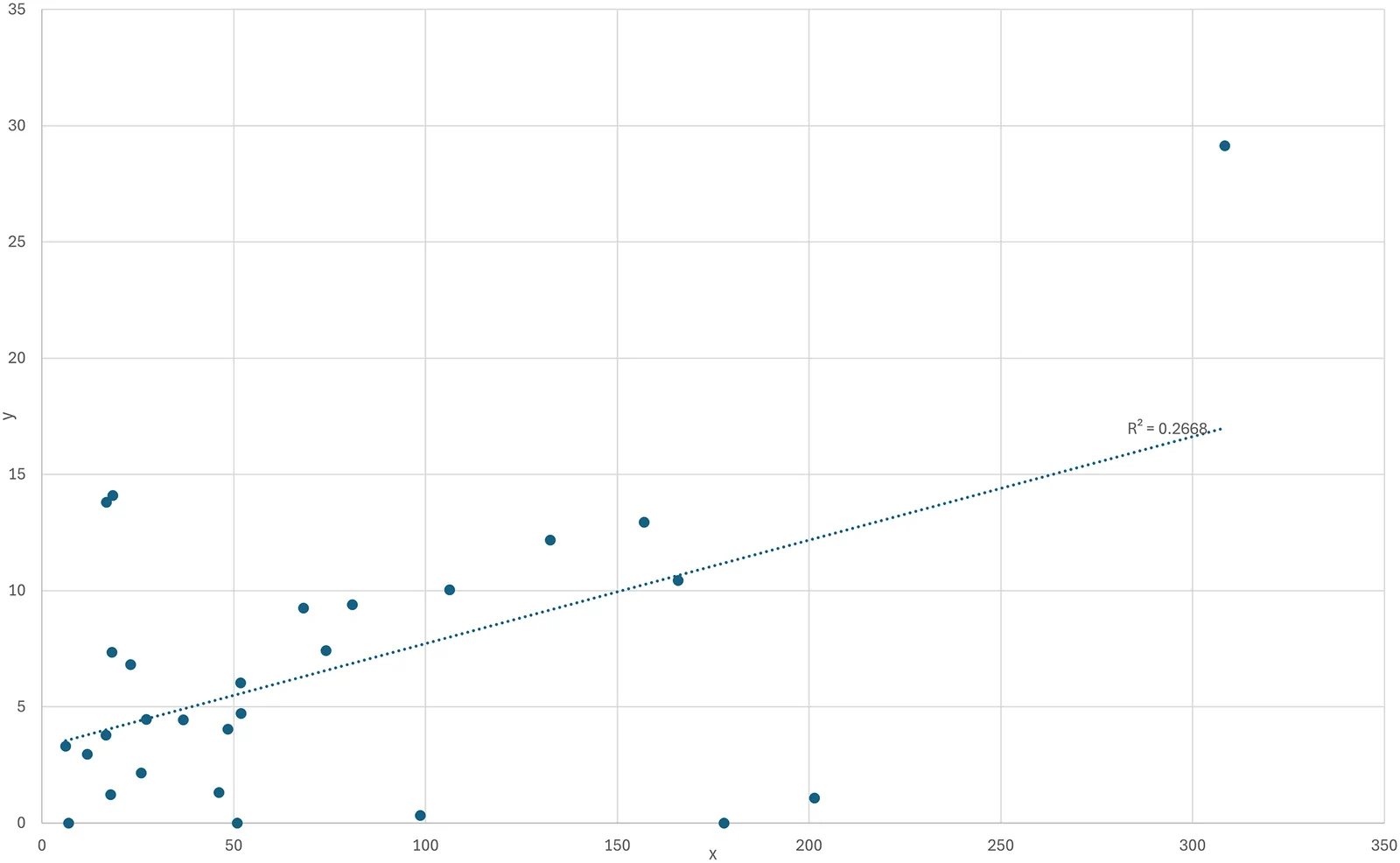
Correlation between mumps (x) and encephalitis lethargica cases (y) in Sweden 1920, Pearsons R2. Figure 7. long description.

Similar to other regions, mumps spread more extensively in Norrköping during the winter months of January to March 1920, accounting for 154 out of 180 reported cases. In contrast, Västerbotten experienced an increase in reported mumps cases from July through December 1920, followed by a rise in reported EL cases during the early winter months of 1921.

National newspapers in early 1921 described an epidemic form of mumps, reportedly originating in England, with a concentration among conscripts in the Stockholm region (including areas near Norrköping). The outbreak was characterized as highly infectious and interpreted by Swedish physicians as either a complication of the Spanish flu or a distinct mumps epidemic [48].

## Discussion

*It is, of course, possible that the virus of encephalitis is more virulent when living in association with the virus of influenza (symbiosis), or that influenza opens the portals for the entrance of the encephalitis virus, just as it does for the streptococcus and pneumococcus. It may be that the same climatic conditions favour the two viruses. In the association of scarlet fever and diphtheria and of measles and whooping cough we have such a tendency for two diseases to run together* ([20], page 23).

The results of this study reveal that EL spread unevenly across Swedish regions, with cases heavily concentrated in the central and northern parts of the country. At the national level, EL peaked in 1921, although the first reports of EL cases appeared in late 1919. Some findings support the hypothesis of an initial epidemic in larger cities (Malmö and Stockholm), followed by a spread to surrounding and rural areas. Of the total 243 EL cases reported in Västerbotten in 1921, 187 were concentrated in Vilhelmina, making it the most intense reported local outbreak of EL in Sweden. In the months preceding the first reported EL cases in Vilhelmina, the municipality experienced a high incidence of mumps.

The study has not been able to determine the cause of encephalitis lethargica or why the inhabitants of Vilhelmina were affected to such a great extent. However, it may contribute a piece to the intricate puzzle surrounding EL. The monocausal hypothesis, [7] which posits a single pathogen as the cause of EL, is difficult to support. If H1N1 influenza were the sole aetiological agent of EL, one might expect more similar incidence patterns across regions heavily affected by influenza. Furthermore, the observed time lag between H1N1 infection and the onset of EL appears too prolonged for H1N1 to act as a sole trigger. Likewise, mumps does not constitute a convincing single-agent explanation for EL, as it is unlikely that such a pattern would have gone unnoticed had the earliest EL cases in Europe been systematically preceded by mumps infection.

If EL instead had an unknown aetiology, one might speculate whether a ‘super-spreader’ event occurred in Vilhelmina – perhaps during the winter market in December – leading to the simultaneous exposure of a large number of individuals. Once a significant portion of the population had developed immunity – whether through mild or severe infection – and as changes in temperature and weather became less favourable for transmission, EL may have faded away as quietly as it had arrived in late November 1920 when the first suspected (but unreported) EL case was noticed in Vilhelmina [41]. However, this scenario is less plausible, as EL first spread in other countries before reaching Sweden [15], and because previous research has not identified other local hotspots with a significant or comparable EL burden worldwide – something that would have been expected during an epidemic of this magnitude.

More reasonably, and following the dual-hypothesis [20, 23], if EL was preceded by another or other infections that paved the way – by increasing its virulence and potentially triggering an immunological response in an already weakened and/or genetically predisposed population [33] – this study could offer valuable insight. In relation to the influenza/H1N1 hypothesis, rural areas in northern Sweden, including Vilhelmina, were among the most severely affected Swedish regions during the Spanish influenza pandemic of 1918–1919 [49]. In 1920, Vilhelmina experienced a relatively high incidence of influenza (H1N1), followed within a short time frame by a mumps epidemic peaking in December 1920, which may be linked to the more severe mumps epidemic reported in England, Norway, and Stockholm in 1920 [32, 48]. It is plausible that this mumps outbreak – consistent with observations reported by Gundersen in Norway [32] – may have been associated with the near-simultaneous occurrence of encephalitis lethargica cases. This temporal sequence may have contributed to conditions that favoured the emergence of EL in this remote and semi-isolated area of Sweden. In northern Sweden around 1920, a combination of low living standards, undernutrition, limited access to healthcare, geographical isolation, and a high burden of infectious diseases likely increased population vulnerability. These conditions may have facilitated the co-occurrence or rapid succession of epidemics, thereby amplifying the risk of complex disease responses. In this context, it may be considered more plausible that EL represented an immunologically mediated response to the preceding epidemics of H1N1/influenza and a potentially more virulent mumps epidemic – or possibly in conjunction with an additional, as yet unidentified pathogen – rather than a condition attributable to a single aetiological agent.

Following this mumps epidemic in Vilhelmina during 1920, it cannot be excluded that some EL cases reported in early 1921 may have represented neurological complications following mumps infection. It is also conceivable that some of the reported EL cases in 1921 actually had a pertussis infection. This might help explain the fact that upper respiratory tract symptoms were more prevalent among the Vilhelmina cases compared to other EL cases reported nation- and worldwide [15]. The fact that mumps was the only reported infection in Vilhelmina from July to December 1920 – suggesting a possible reporting discrepancy or data lag for the other infections studied here. Although these factors alone cannot fully account for the pronounced outbreak of encephalitis lethargica in Vilhelmina during the winter of 1921, the meticulous investigation and verifying of EL cases conducted by physicians Kling and Liljenquist [41] – who had been engaged in sustained research and experimental work on EL since December 1919 – remains noteworthy. At that time, few individuals in Sweden possessed a comparable level of expertise regarding the disease.

Finally, the autoimmune component of EL is an intriguing and promising hypothesis [7, 9, 10, 13, 35], where discoveries of autoantibodies mistakenly targeting the central nervous system represent a rapidly emerging field of research [50, 51]. While EL does not fit a specific autoimmune encephalitis (such as *NMDA* receptor encephalitis), it fits the *general pattern* of autoimmune encephalitis including cognitive decline and memory deficits, altered mental status, sleep disturbances, psychiatric alterations, and, in later stages, parkinsonism [15, 50]. Further, it connects the historical EL epidemic with the current context such as Post-COVID syndromes. In this light, several pieces of the puzzle for future and similar epidemics might be gathering in the shadows surrounding encephalitis lethargica.

### Strengths and shortcomings

A key strength of this study lies in the availability and utilization of national statistics from Swedish authorities, including annual reports on the number of individuals affected by encephalitis lethargica by region. The Annual Reports of the Chief Provincial Doctors provided further in-depth insights into how EL and other infectious diseases affected the population at the local level. By bringing together historical records such as these, a detailed picture of the disease panorama emerges – adding valuable knowledge about the epidemiological milieu in which EL proliferated. The lack of official reported data on mumps from 1921 on the national and regional levels represents a significant limitation. It is possible that, as in Vilhelmina, mumps may have infected the population during the preceding winter months of 1920, with encephalitis lethargica following in the first 1–3 months of 1921. In making such comparisons, it is necessary – as done in this study – to break down the regions into smaller units, such as at the parish level, and identify locations where EL appears to have gained a firm foothold in the population. Following the result presented in this study, to find another Swedish parish with an incidence rate in parity with Vilhelmina will not be feasible. In this regard, however, Kling and Liljenquist [41] were possibly correct: Vilhelmina did constitute a ‘virgin land’ for the study of encephalitis lethargica – at least within a Swedish context.

Finally, an additional consideration is that the Vilhelmina outbreak received considerable contemporary attention. The detailed field investigations undertaken by Kling and Liljenquist after the outbreak started may have improved case detection, particularly of mild or abortive cases, resulting in more complete reporting than in many other Swedish districts.

## Data Availability

The data that support the findings of this study are available from the corresponding author upon reasonable request.

## Long descriptions

### Figure 2 Long description

The Y-axis is labeled Number of cases with increments of 200 from 0 to 1600. The X-axis is labeled Year and lists every year from 1920 to 1945. The data line begins in 1920 at approximately 350 cases. It rises sharply to a global peak of just over 1500 cases in 1921. In 1922, it drops precipitously to around 200 cases before a secondary peak of approximately 530 cases in 1923. From 1924 onward, the trend shows a steady, non-linear decline. By 1925, cases drop to 200, and by 1930, they reach approximately 120. The line continues to flatten toward the baseline, staying below 100 cases from 1937 through 1945, with a minor negligible uptick in 1942.

Navigate back to Figure 2.

### Figure 3 Long description

A series of 12 maps of Sweden, each representing a year from 1919 to 1930. Each map uses a color gradient from dark blue (low incidence) to yellow (high incidence) to show cases per 100,000 people by administrative region.

* 1919: Low incidence nationwide with small yellow hotspots on the Southeast and Southwest coasts. Scale 0 to 8.

* 1920: Incidence increases slightly in the South. Scale 0 to 20.

* 1921: Significant peak in the North and Central regions with yellow shading indicating incidence up to 125.

* 1922: Incidence drops significantly, with a small yellow area in the Southwest. Scale 0 to 10.0.

* 1923: Moderate incidence in Central Sweden. Scale 5 to 20.

* 1924: High incidence concentrated in the West-Central region. Scale 0 to 30.

* 1925: Moderate incidence in the West-Central region. Scale 0.0 to 7.5.

* 1926: Low to moderate incidence spread across Central and Southern regions. Scale 0.0 to 10.0.

* 1927: Widespread moderate incidence, particularly in the North and Central regions. Scale 0 to 5.

* 1928: Incidence concentrated in the Southern tip. Scale 0.0 to 12.5.

* 1929: Low incidence nationwide with a small yellow area in the South. Scale 0 to 8.

* 1930: Very low incidence across all regions. Scale 0 to 9.

Red circles highlight specific coastal urban centers across all maps. The data source is cited as Sveriges officiella statistik, Allman hälso och sjukvård, 1920-1930.

Navigate back to Figure 3.

### Figure 4 Long description

The vertical y-axis is labeled Number of cases with increments of 100 ranging from 0 to 600. The horizontal x-axis is labeled Month and lists the twelve months from Jan to Dec. The data is represented by dark teal vertical bars.

* Jan has the highest peak at approximately 520 cases.

* Feb shows a slight decrease to approximately 450 cases.

* March shows a significant drop to approximately 240 cases.

* April continues the downward trend to approximately 105 cases.

* May drops to approximately 70 cases.

* June drops to approximately 45 cases.

* July drops to approximately 25 cases.

* August reaches a low of approximately 15 cases.

* Sep shows a minor uptick to approximately 25 cases.

* Oct drops to approximately 10 cases.

* Nov shows the lowest point with approximately 5 cases.

* Dec ends the year with a small increase to approximately 15 cases.

Navigate back to Figure 4.

### Figure 5 Long description

The X axis is labeled Month, ranging from January 1920 to December 1921. The Y axis is labeled Number of cases, with increments of 250 up to 750. A legend on the right identifies seven diseases: Diphtheria (teal), Encephalitis lethargica (yellow), Influenza (purple), Measles (pink), Mumps (orange), Scarlet fever (brown), and Whooping cough (green).

Data trends:

* The most prominent feature is a massive spike in Influenza cases in February and March 1920, peaking at nearly 900 total cases in March.

* A second, smaller wave of mixed infections occurs between September 1920 and March 1921, peaking in November 1920 at approximately 400 cases.

* Diphtheria remains present throughout most of the period, appearing as the top layer in many bars.

* Encephalitis lethargica shows a notable presence specifically in February and March 1921.

* Measles and Mumps cases are concentrated in the late 1920 to early 1921 period.

* Whooping cough and Scarlet fever form the base layers of many bars with relatively low, consistent counts compared to the major outbreaks.

Navigate back to Figure 5.

### Figure 6 Long description

A stacked bar chart titled Disease cases per month in Vilhelmina. The Y axis is labeled Number of cases with increments of 50 up to 150. The X axis is labeled Month and lists every month from Jan 1920 to Dec 1921. A legend on the right identifies six diseases by color.

* Diphtheria (teal) appears in small numbers throughout the period, peaking slightly in Jan 1920 and Mar 1921.

* Influenza (purple) shows a significant wave from Feb 1920 to May 1920, peaking at approximately 50 cases in Mar 1920.

* Mumps (orange) shows a large, sustained outbreak from Jul 1920 to Dec 1920, increasing steadily each month to a peak of over 110 cases in Dec 1920.

* Encephalitis lethargica (yellow) appears as a massive, sharp spike in early 1921, reaching the chart maximum of 150 cases in Feb 1921 and approximately 65 cases in Mar 1921.

* Whooping cough (green) appears in early 1921, with about 10 to 15 cases per month from Jan to Apr.

* Measles (pink) is barely visible with a negligible count in Nov 1921.

The chart shows three distinct epidemic waves: Influenza in Spring 1920, Mumps in Autumn 1920, and Encephalitis lethargica in Winter 1921.

Navigate back to Figure 6.

### Figure 7 Long description

The scatter plot uses a horizontal x-axis ranging from 0 to 350 in increments of 50 and a vertical y-axis ranging from 0 to 35 in increments of 5.

Data Distribution:

* Most data points are clustered in the lower-left quadrant between x values of 0 and 100 and y values of 0 and 15.

* A single significant outlier is located at the far top-right of the plot near x equals 310 and y equals 29.

* Several points lie directly on the x-axis (y equals 0) at approximately x equals 10, 50, 100, and 180.

Trend Line:

* A dotted linear regression line starts at approximately y equals 3.5 on the y-axis and rises steadily toward the right.

* The line terminates near the outlier at approximately x equals 310 and y equals 17.

* A text label placed above the right end of the trend line reads R super 2 equals 0.2668.

Navigate back to Figure 7.
